# Supplementary figures and images for: Acetylation-mediated remodeling of the nucleolus regulates cellular acetyl-CoA responses
Source: PLoS Biol. 2020 Nov 30;18(11):e3000981. doi: 10.1371/journal.pbio.3000981 (PMC7728262; doi:10.1371/journal.pbio.3000981)

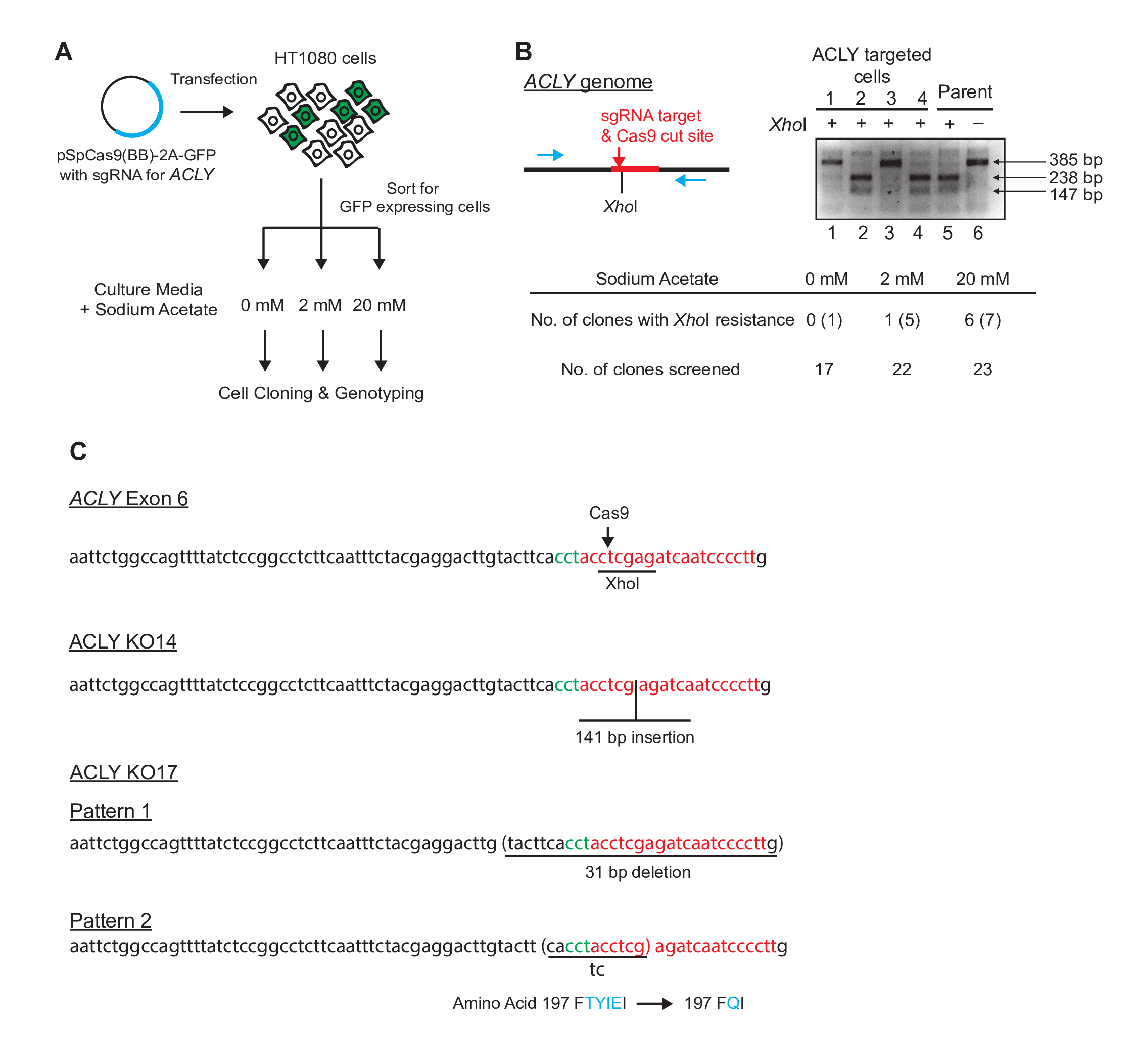

Supplement: S1 Fig — (A) Schema of genome editing for ACLY. A GFP-tagged sgRNA/Cas9 plasmid targeting ACLY was transfected into HT1080 cells. Following cell sorting by flow cytometry for GFP positive transfected cells, cells were recovered in either standard medium (0-mM added acetate) or 2- or 20-mM sodium acetate–supplemented media. Recovered cell clones were genotyped to identify genome editing for ACLY as in “B”. (B) Schema of genotyping for ACLY genome-edited cell clones and representative genotyping results (top left). The sgRNA-targeted and Cas9 cleavage site in ACLY exon 6, which contains a single XhoI site overlapped with a Cas9 cleavage site, was PCR amplified by flanked primers (blue). XhoI digestion of the PCR product (385 bp) provided 238 bp and 147 bp fragments when the XhoI site was intact (top right). XhoI resistance was indicative of insertion or deletion (in/del) by CRISPR-mediated non-homologous end joining (NHEJ). Numbers of clones that exhibited complete (or partial) XhoI resistance and numbers of clones screened were shown for each culture media condition (bottom). (C) The sequence of exon 6 in wild-type ACLY and the edited sequences in ASA-KO14 and KO17 cells. The sgRNA target site (red), PAM motif (green), Cas9 cut site (arrow head), XhoI site, and detected in/del were indicated. We detected only a single pattern of insertion in ASA-KO14 cells. Note that 1 allele of ASA-KO17 possesses an indel, which causes a frame shift mutation replacing amino acids “T-Y-I-E” with “Q”. This might explain the detected ACLY peptides in the TMT proteomics using ASA-KO17 cells (S3 Table). (TIF) [file pbio.3000981.s001.tif]

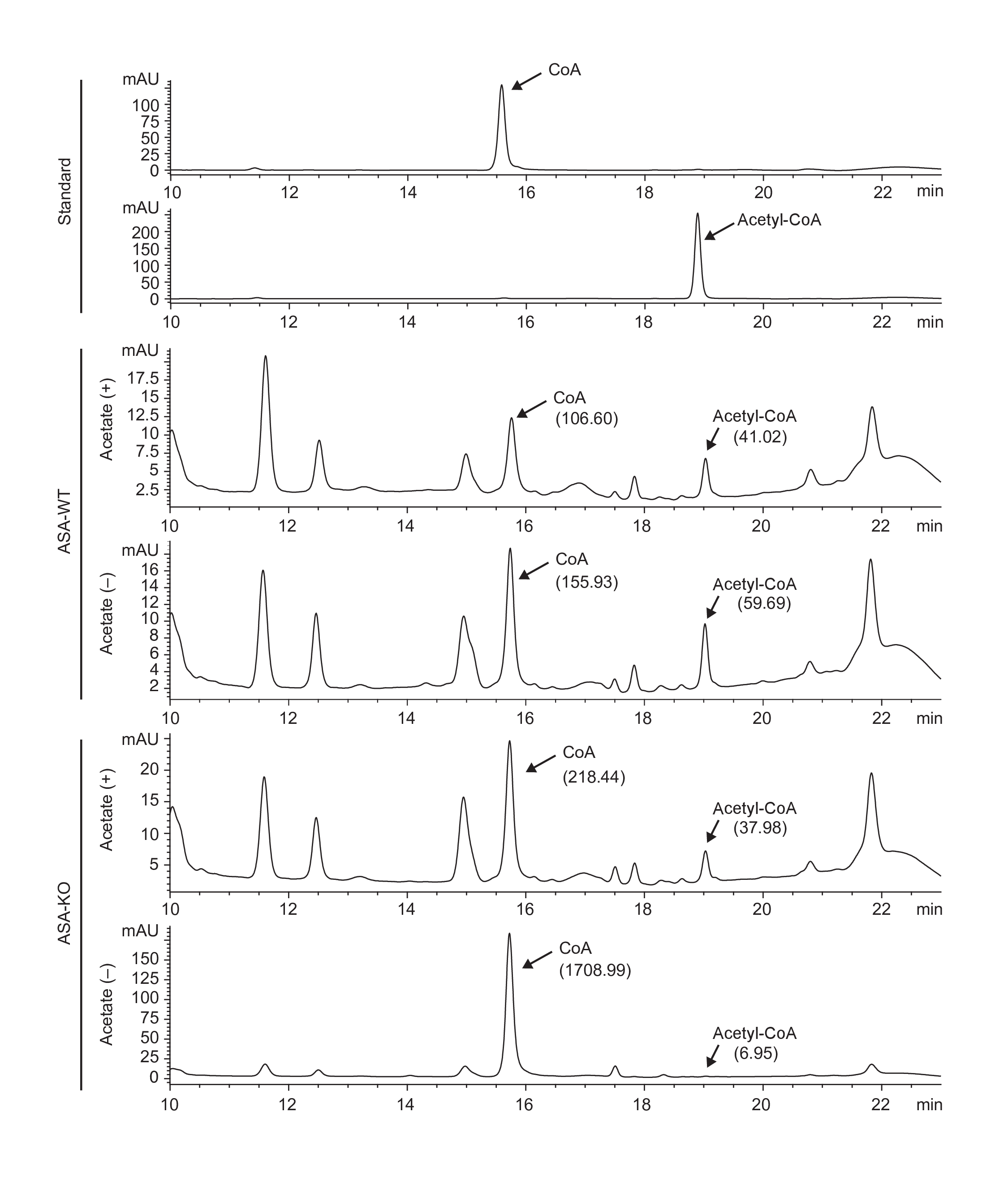

Supplement: S2 Fig — HPLC UV chromatograms of cell lysates from a representative experiment for acetyl-CoA and CoA quantification shown in Fig 1E. Standard acetyl-CoA (100 μM) and CoA (100 μM) run in the same experiment are also shown. Values in parentheses indicate the peak area. (TIF) [file pbio.3000981.s002.tif]

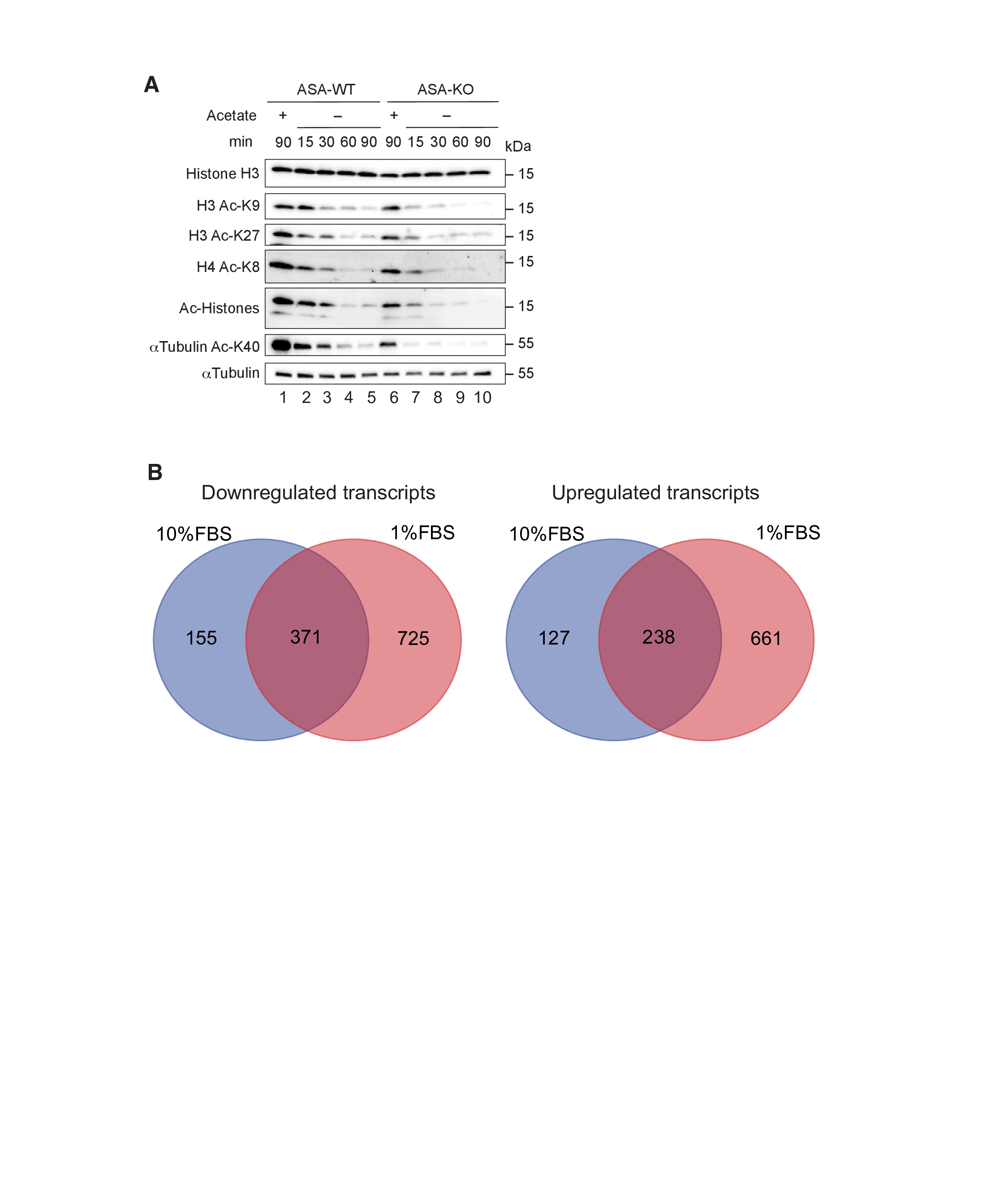

Supplement: S3 Fig — (A) Immunoblotting for the indicated acetylated and total protein levels in ASA-WT and ASA-KO cells cultured in 1% FBS containing media with or without 20-mM acetate for the indicated times. (B) Venn diagram for down-regulated transcripts {log2 FC [(−) Acetate / (+) Acetate] < −1.0. q<0.05} and up-regulated transcripts {log2 FC [(−) Acetate / (+) Acetate] >1.0. q<0.05} in the RNA sequencing with the 10% and 1% FBS conditions (S2 Table). (TIF) [file pbio.3000981.s003.tif]

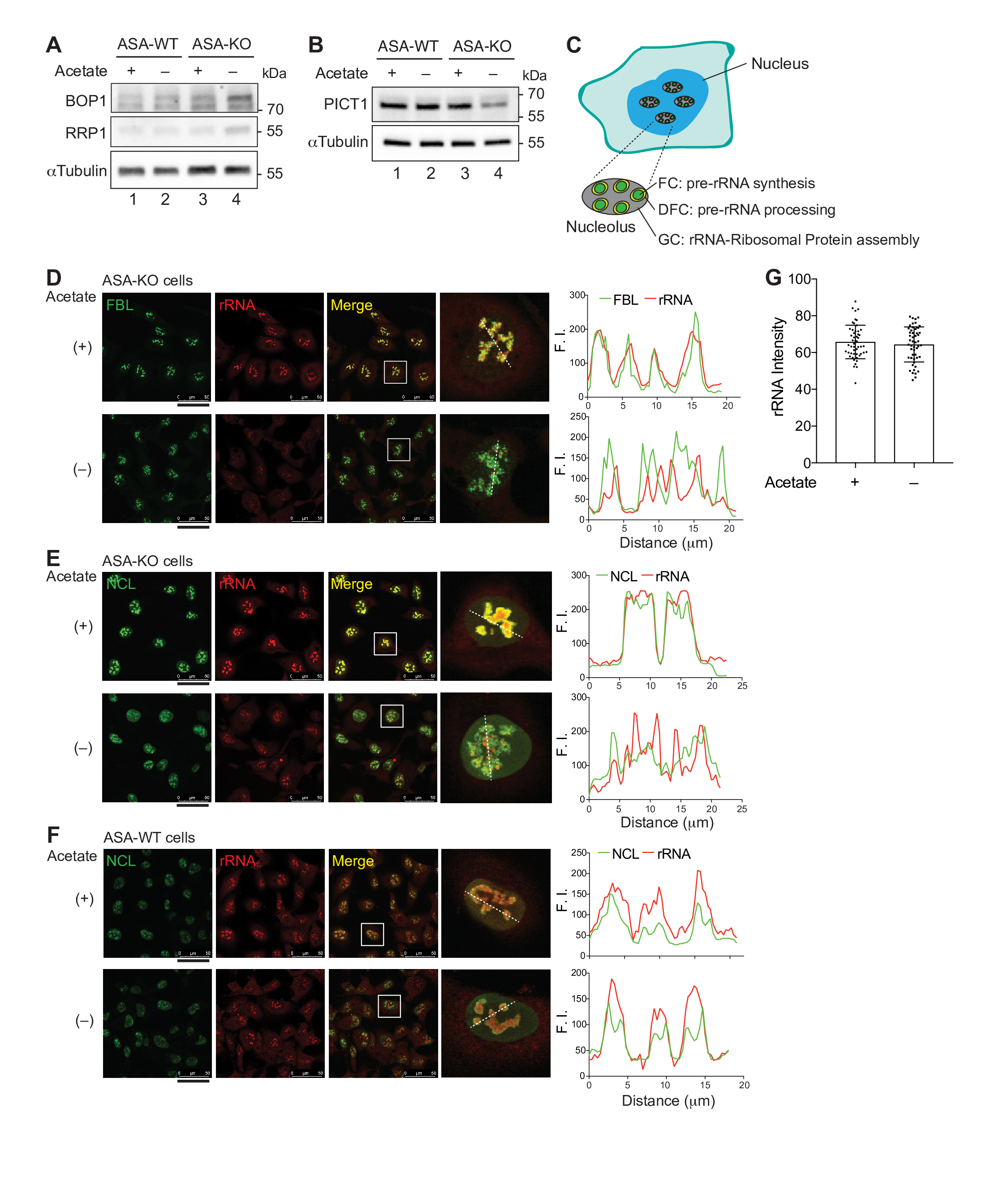

Supplement: S4 Fig — (A and B) Immunoblotting for nucleolar protein BOP1 and RRP1 (A) and PICT1 (B) in ASA-WT and ASA-KO cells cultured in 10% or 1% FBS containing media with or without acetate for 4 hours. αTubulin was used as a loading control. (C) Schematic representation of the nucleolus consisting of the fibrillar center (FC; green), the dense fibrillar component (DFC; yellow), and the granular component (GC; gray). (D–F) Immunostaining for FBL (D) and NCL (E and F) along with rRNA staining in ASA-KO (D and E) and ASK-WT (F) cells cultured in 1% FBS containing media with or without acetate for 4 hours. The scale bars under the left images indicate 50 μm. Magnified nuclear images (surrounded by a white square) are shown. Line profiles for indicated fluorescent intensities (FI) determined along the white dashed lines are shown to the right. (G) Quantification of the mean fluorescent intensity of the rRNA signal per nucleus in “F.” Data are shown as mean ± SD (n = 51 cells per condition). The data underlying the graphs in S4 Fig can be found in S1 Data. (TIF) [file pbio.3000981.s004.tif]

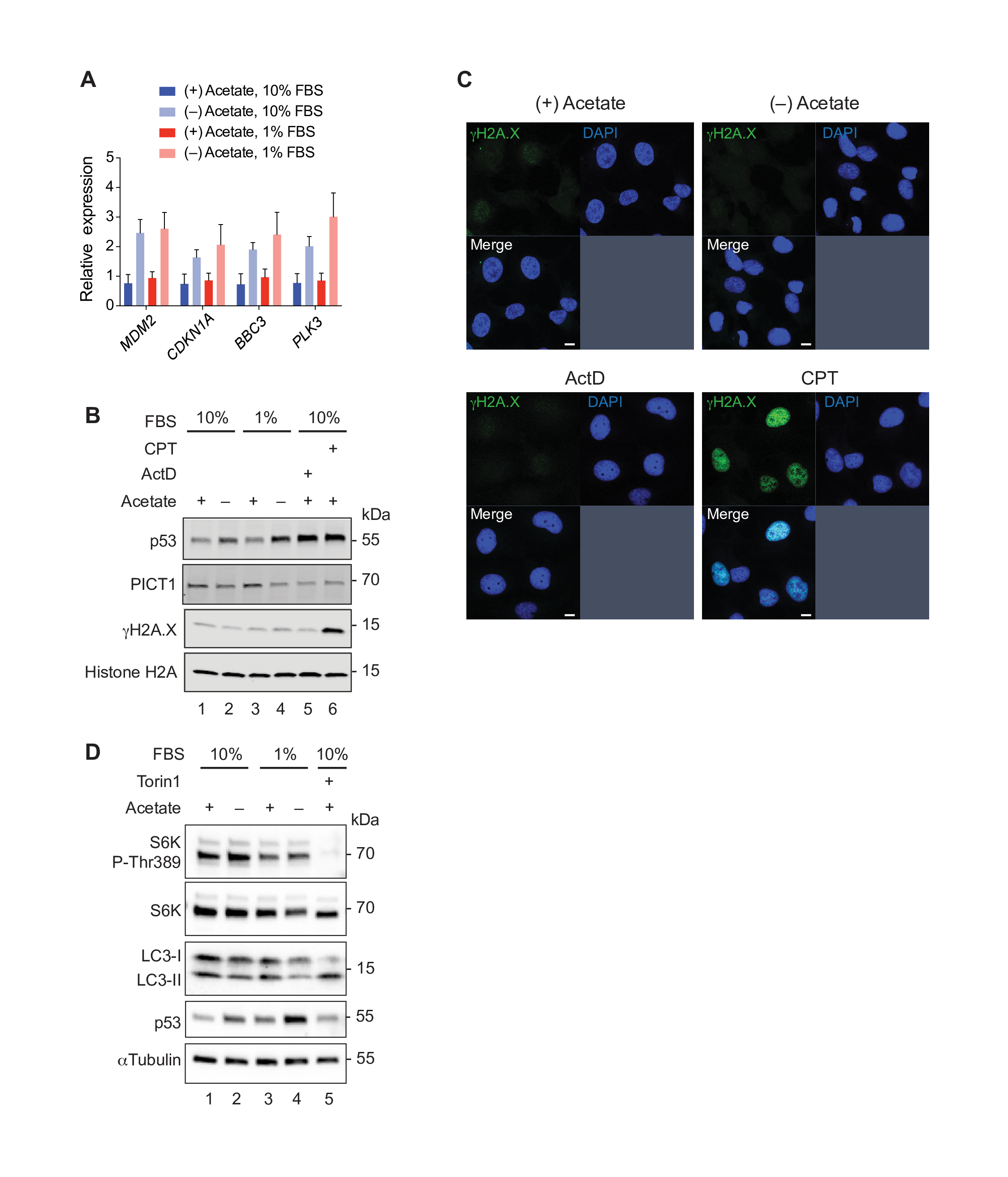

Supplement: S5 Fig — (A) Relative mRNA expressions of p53 target genes selected from the RNA sequencing in S2 Table. Data are shown as mean ± SD (n = 3 biological replicates). (B) Immunoblotting for levels of p53, PICT1, γH2A.X, and histone H2A in ASA-KO cells cultured in 10% or 1% FBS containing media with or without acetate for 4 hours, or treated with 5-nM ActD or 1-μM Camptothecin (CPT), a DNA damage inducer, for 4 hours. (C) Immunostaining for γH2A.X in ASA-KO cells cultured in the same conditions as in “A.” The scale bars indicate 10 μm. (D) Immunoblotting for levels of phosphorylated Threonine 389 of S6 kinase (S6K), total S6K, LC3, and p53 in ASA-KO cells cultured in 10% or 1% FBS containing media with or without acetate, or treated with 1-μM Torin 1 for 4 hours. The data underlying the graphs in S5 Fig can be found in S1 Data. (TIF) [file pbio.3000981.s005.tif]

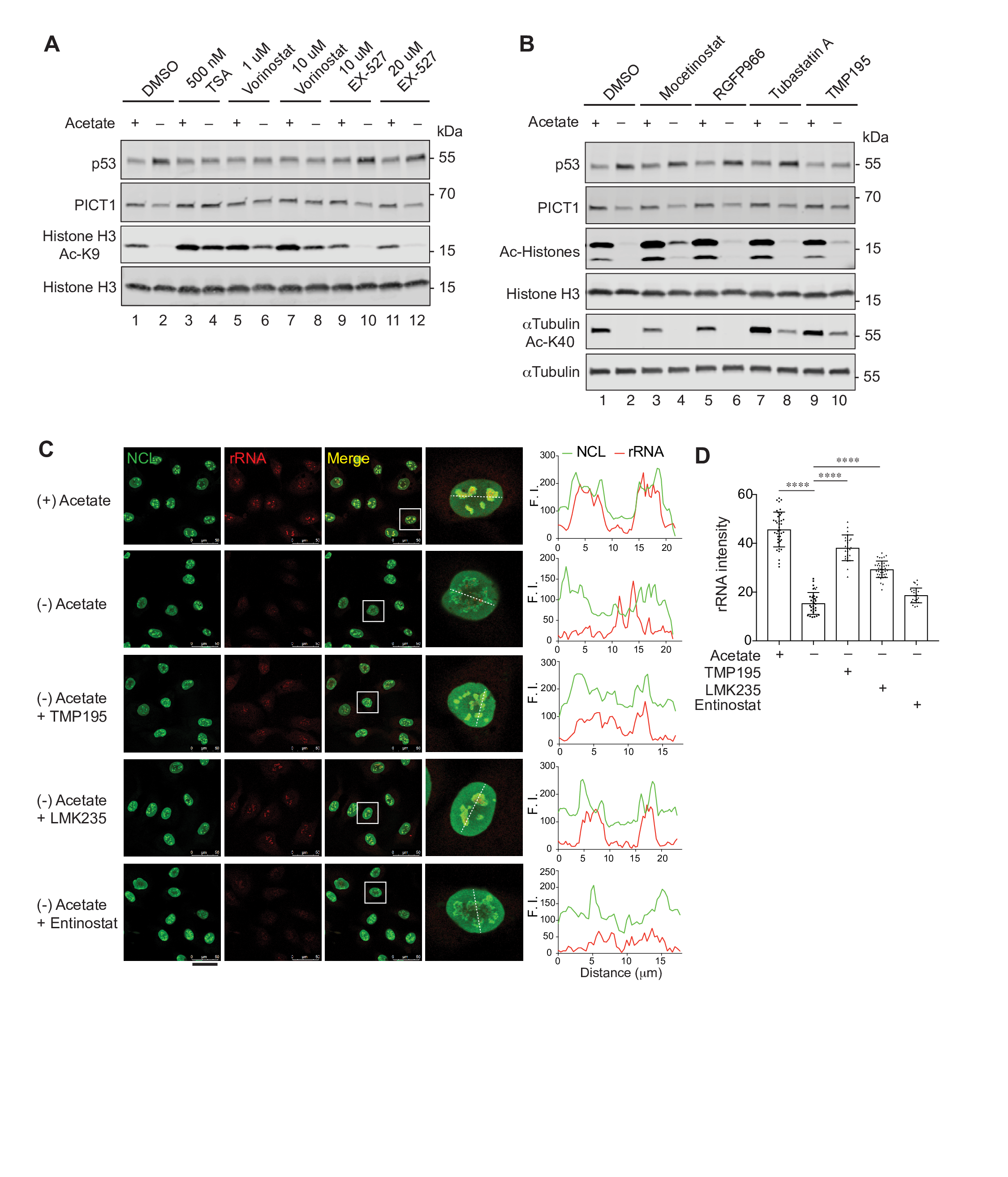

Supplement: S6 Fig — (A) Immunoblotting for the indicated proteins in ASA-KO cells cultured in 1% FBS containing media with or without acetate, and in the presence or absence of indicated HDAC inhibitors for 4 hours. (B) Immunoblotting for indicated proteins in ASA-KO cells cultured in 1% FBS containing media with or without acetate, and in the presence or absence of indicated HDAC inhibitors (10 μM) for 4 hours. (C) Immunostaining for NCL along with rRNA dye staining in ASA-KO cells cultured in 1% FBS containing media with or without acetate and in the presence or absence of indicated HDAC inhibitors (50-μM TMP195, 10-μM LMK, or 50-μM Entinostat) for 4 hours. The scale bar under the left images indicates 50 μm. Magnified nuclear images (surrounded by a white square) are shown. Line profiles for indicated fluorescent intensities (FI) determined along the white dashed lines are shown to the right. (D) Quantification of the mean fluorescent intensity of the RNA signal per nucleus in (C). Data are shown as mean ± SD (n = 29 to 53 cells per condition). ****P < 0.0001 (1-way ANOVA followed by Tukey multiple comparisons test). The data underlying the graphs in S6 Fig can be found in S1 Data. (TIF) [file pbio.3000981.s006.tif]

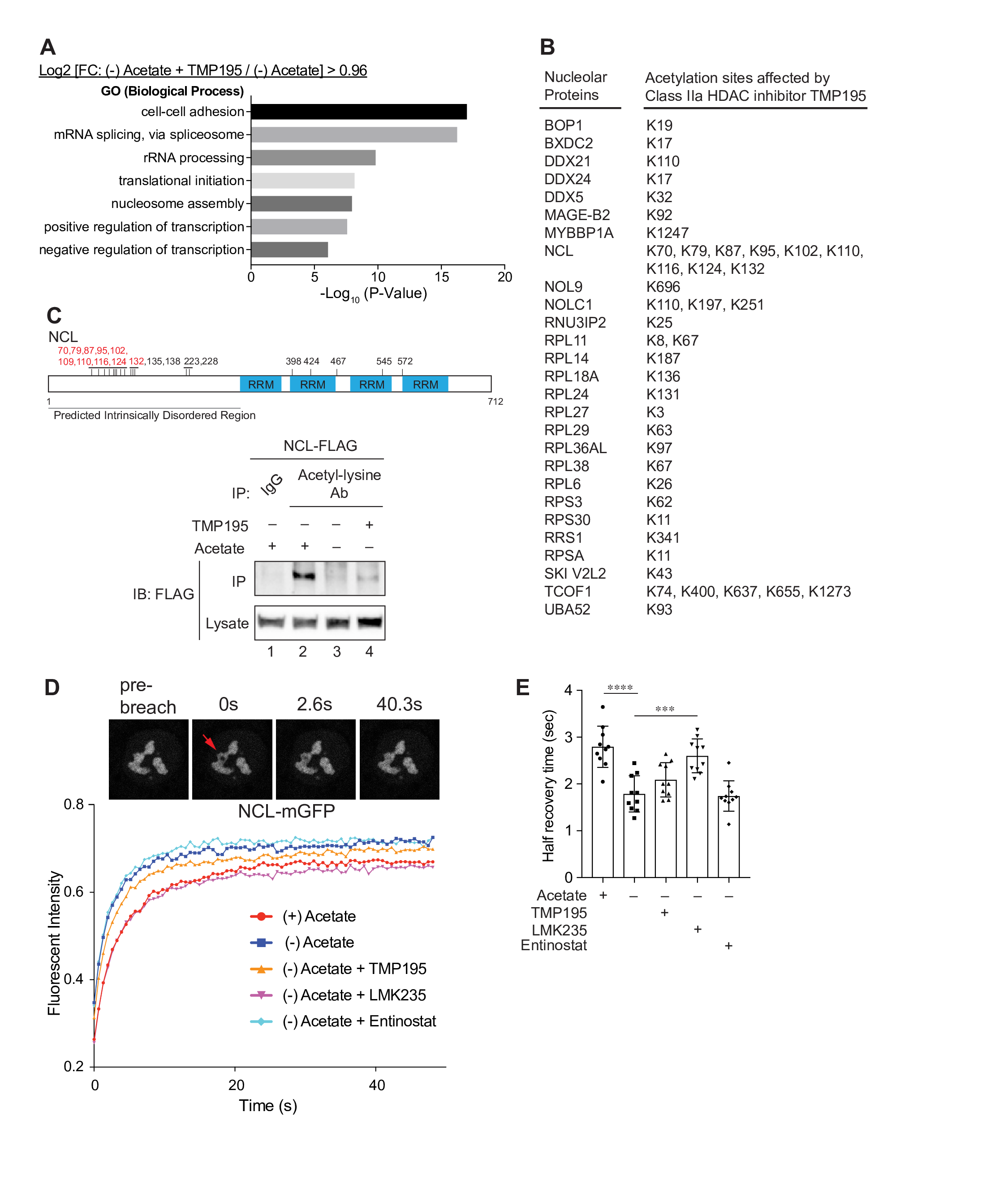

Supplement: S7 Fig — (A) Gene ontology (GO) analysis for TMP195-sensitive 365 acetylated peptides {S1 Table, column C, Log2 [FC: (−) Acetate + TMP195 / (−) Acetate] >0.96}. Enriched representative biological processes are shown. (B) List of selected nucleolar proteins from S1 Table as in “A,” and their acetylation sites affected by TMP195. (C) Schematic representation of acetylation sites in NCL (top). TMP195-sensitive acetylation sites are shown in red. RRM, RNA recognition motif. Immunoprecipitation with the acetyl-lysine motif antibody and immunoblotting for NCL-3xFLAG in ASA-KO cells cultured in 1% FBS containing media with or without acetate, and in the presence or absence of 50-μM TMP195 for 90 minutes (bottom). (D) FRAP of NCL-mGFP in ASA-KO cells cultured in 1% FBS containing media with or without acetate, and in the presence or absence of 50-μM TMP195, 10-μM LMK235, or 50-μM Entinostat for 90 minutes. Data are shown as mean (n = 10 independent cells from 2 independent experiments). Representative nucleolar images expressing NCL-mGFP before and after photobleaching are shown on the top. The red allow indicates the ROI. (E) Half recovery times (sec) obtained from the FRAP curves in “D.” Data are shown as mean ± SD (n = 10 independent cells). ***P < 0.001, ****P < 0.0001 (1-way ANOVA followed by Tukey multiple comparisons test). The data underlying the graphs in S7 Fig can be found in S1 Data. (TIF) [file pbio.3000981.s007.tif]

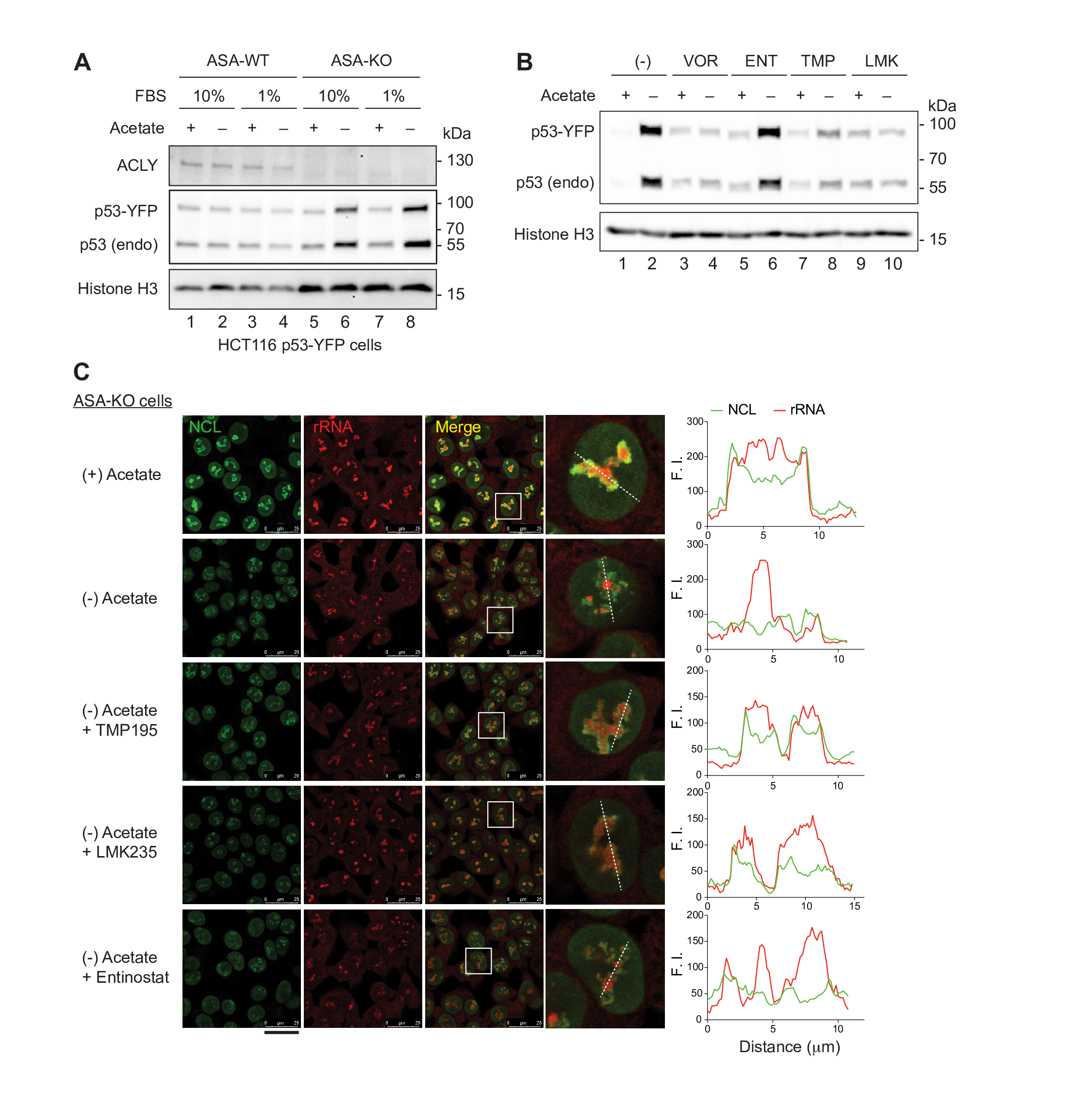

Supplement: S8 Fig — (A) Immunoblotting for ACLY and p53 levels in HCT116 p53-YFP ASA-WT and ASA-KO cells cultured in 10% or 1% FBS containing media with or without acetate for 4 hours. Histone H3 is shown as a loading control. (B) Immunoblotting for p53 levels in HCT116 p53-YFP ASA-KO cells cultured in 1% FBS containing media with or without acetate, and in the presence or absence of indicated HDAC inhibitors for 4 hours. Following concentrations of HDAC inhibitors were used: 10-μM Vorinostat (VOR), 70-μM Entinostat (ENT), 50-μM TMP195 (TMP), and 10-μM LMK235 (LMK). (C) Immunostaining for NCL along with rRNA dye staining in ASA-KO cells cultured in 1% FBS containing media with or without acetate, and in the presence or absence of indicated HDAC inhibitors (50-μM TMP195, 10-μM LMK, or 50-μM Entinostat) for 4 hours. The scale bar under the left images indicates 50 μm. Magnified nuclear images (surrounded by a white square) are shown. Line profiles for indicated fluorescent intensities (FI) determined along the white dashed lines are shown to the right. The data underlying the graphs in S8 Fig can be found in S1 Data. (TIF) [file pbio.3000981.s008.tif]
