# Supplementary material for: Acetylation-mediated remodeling of the nucleolus regulates cellular acetyl-CoA responses
Source: PLoS Biol. 2020 Nov 30;18(11):e3000981. doi: 10.1371/journal.pbio.3000981 (PMC7728262; doi:10.1371/journal.pbio.3000981)

Fig 1B

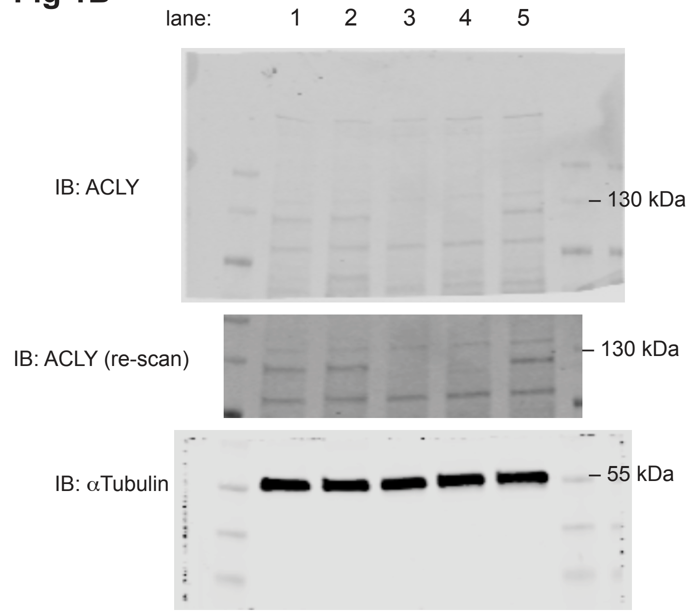

S1B Fig

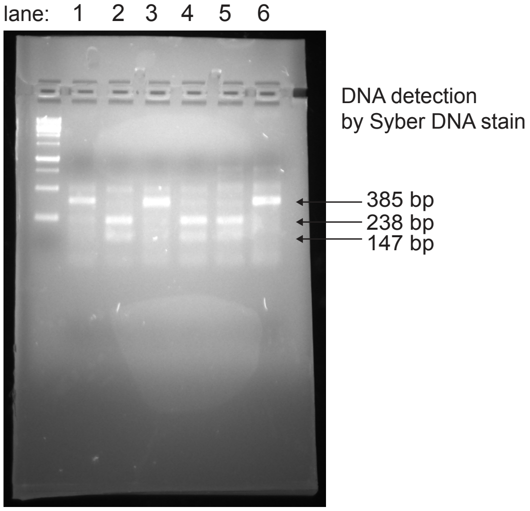

Fig 2D

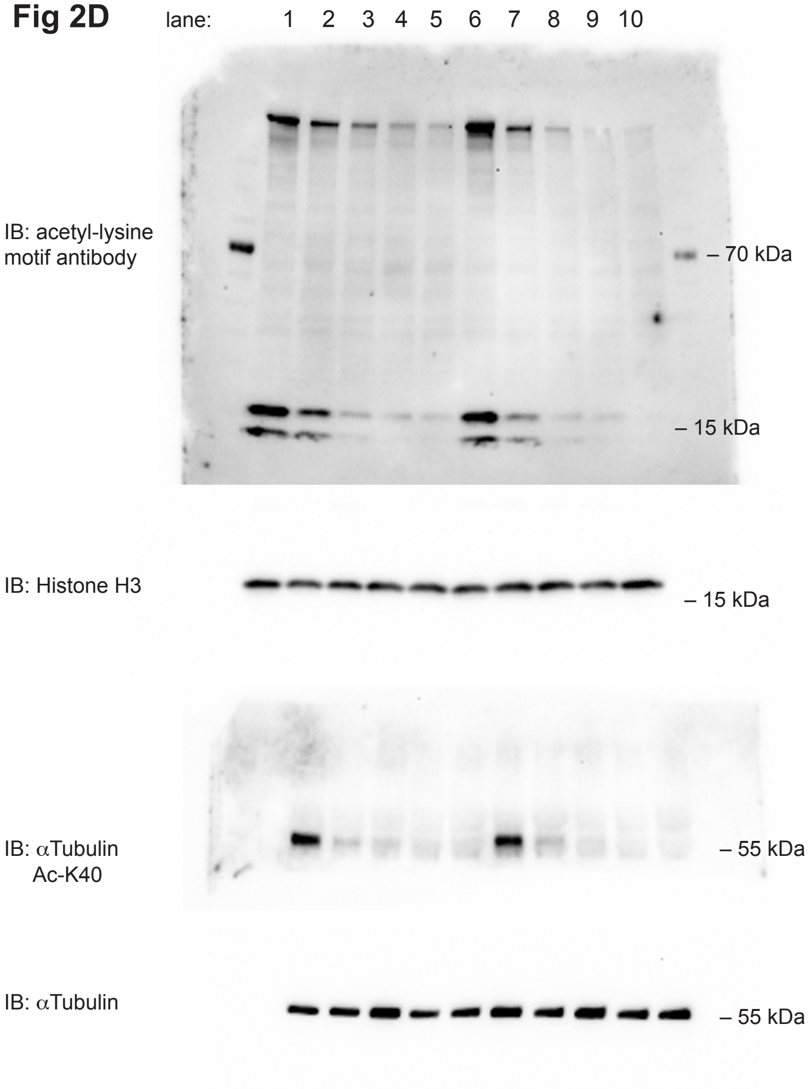

Fig 2E

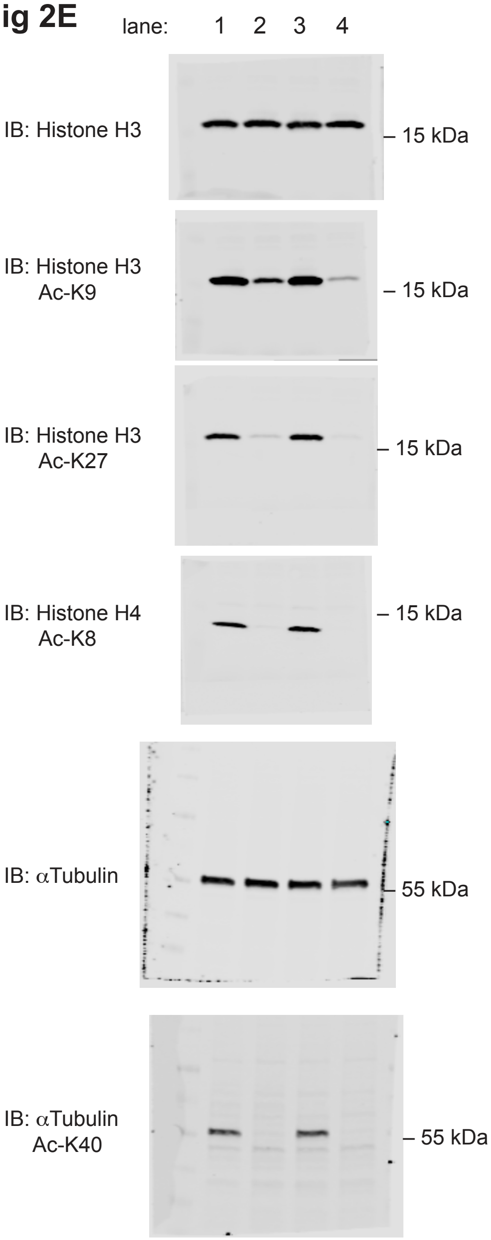

S3A Fig

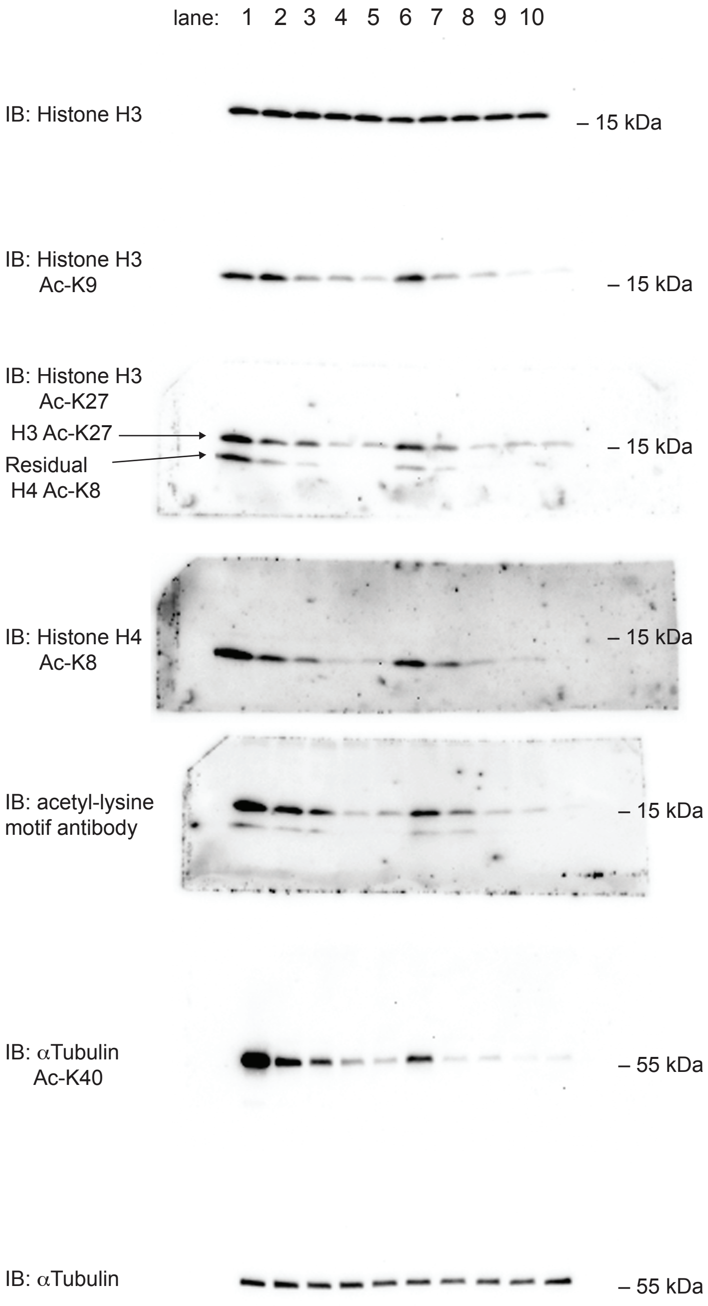

S4A Fig

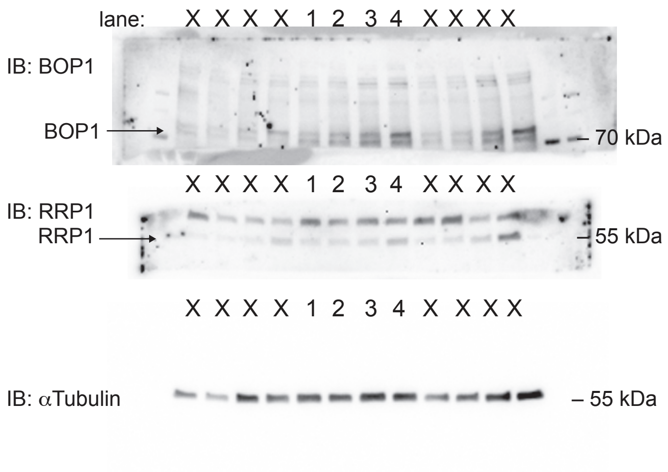

S4B Fig

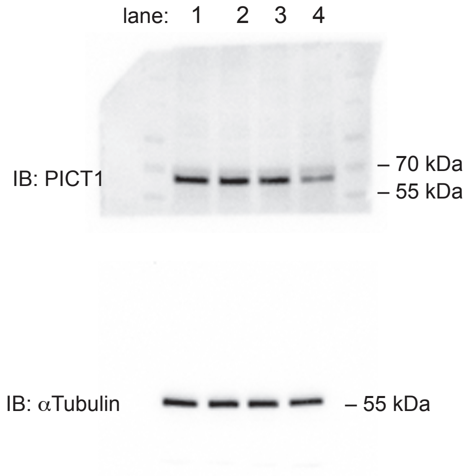

Fig 4A

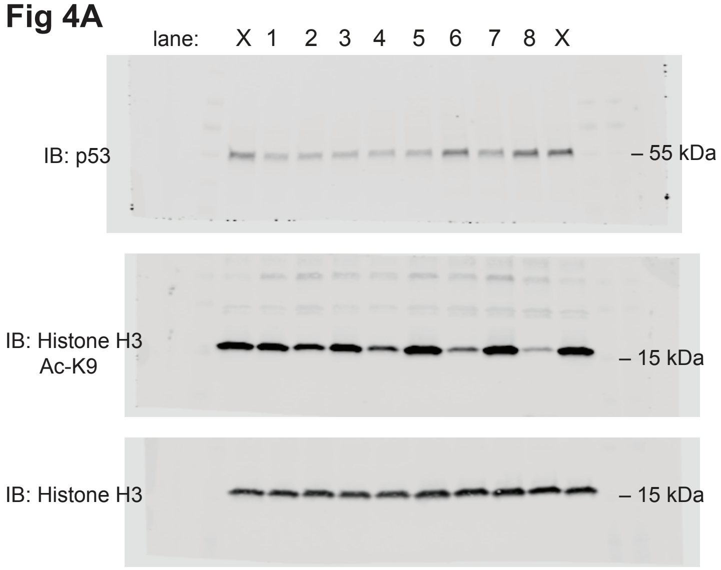

Fig 4C

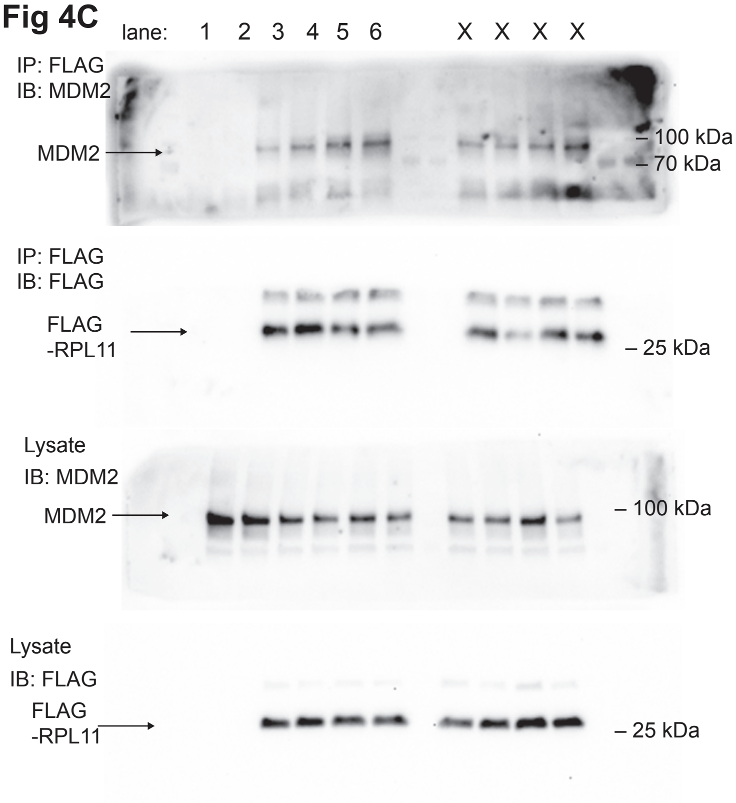

Fig 4D

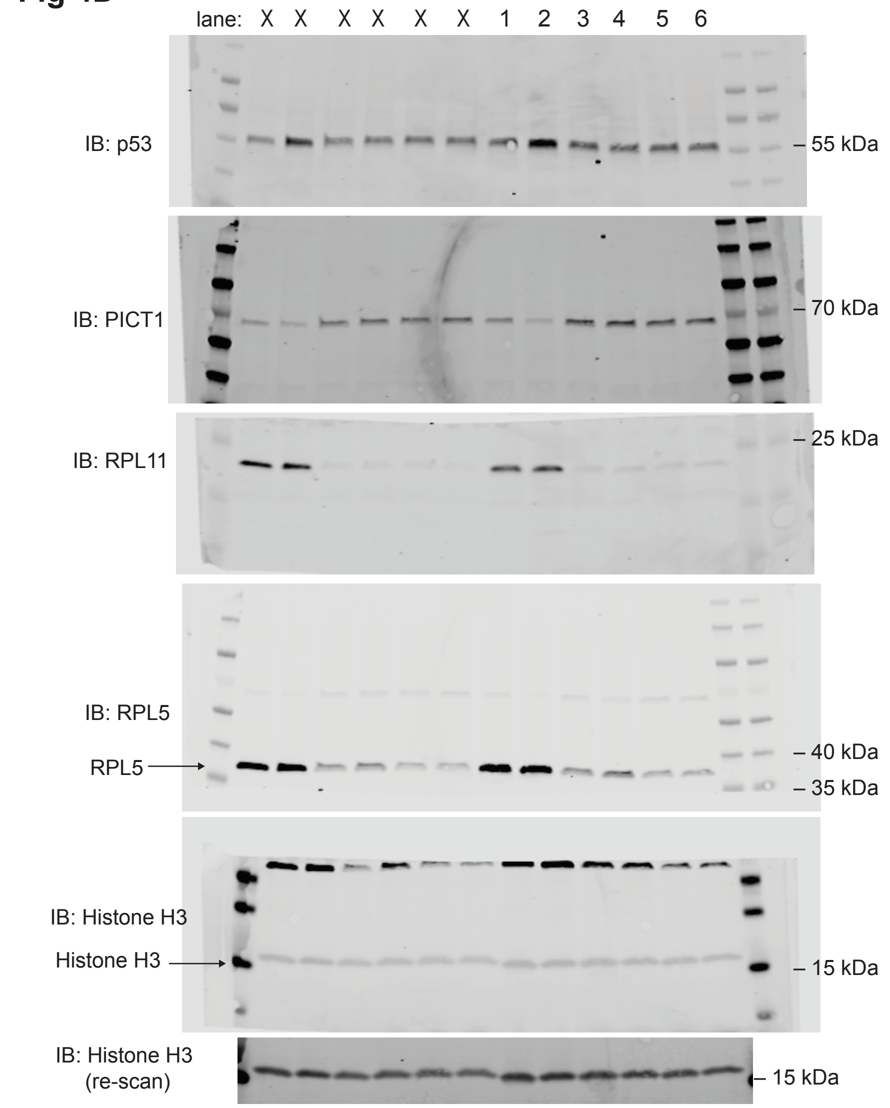

S5B Fig

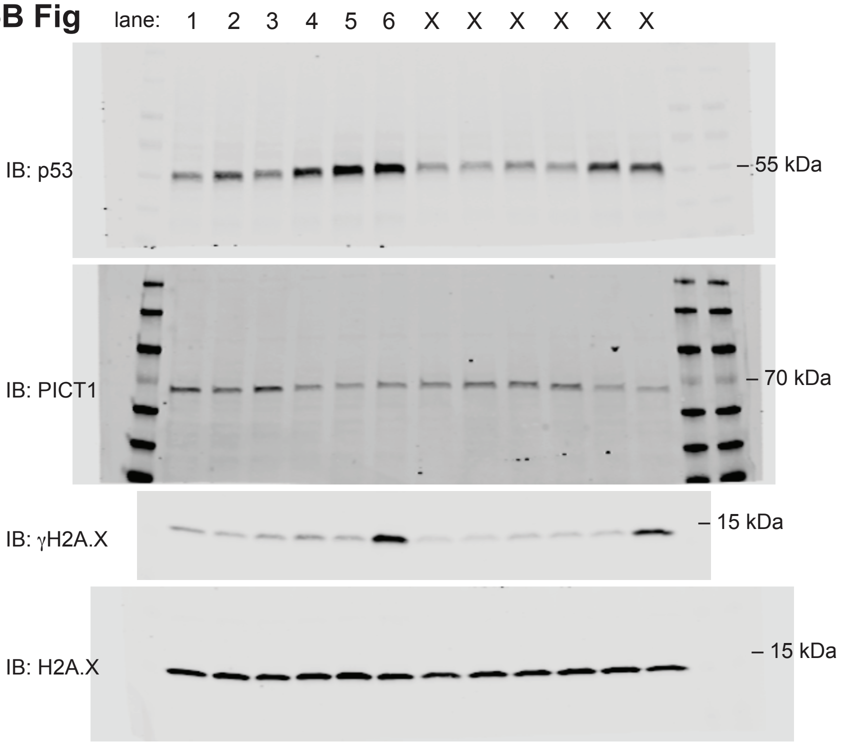

S5D Fig

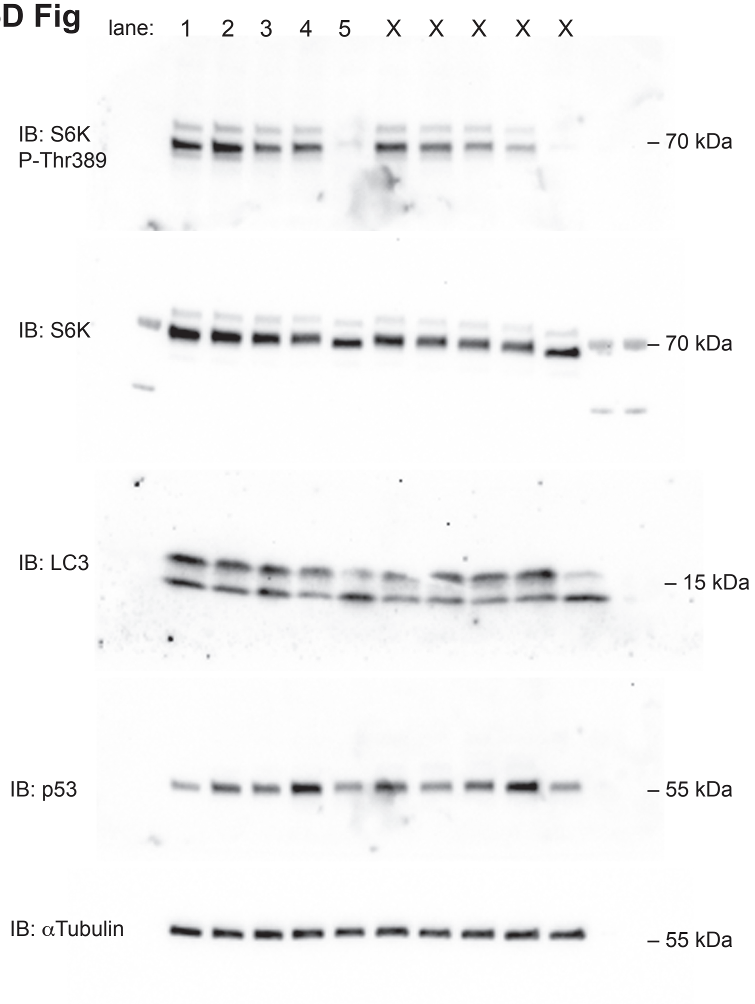

Fig 5B

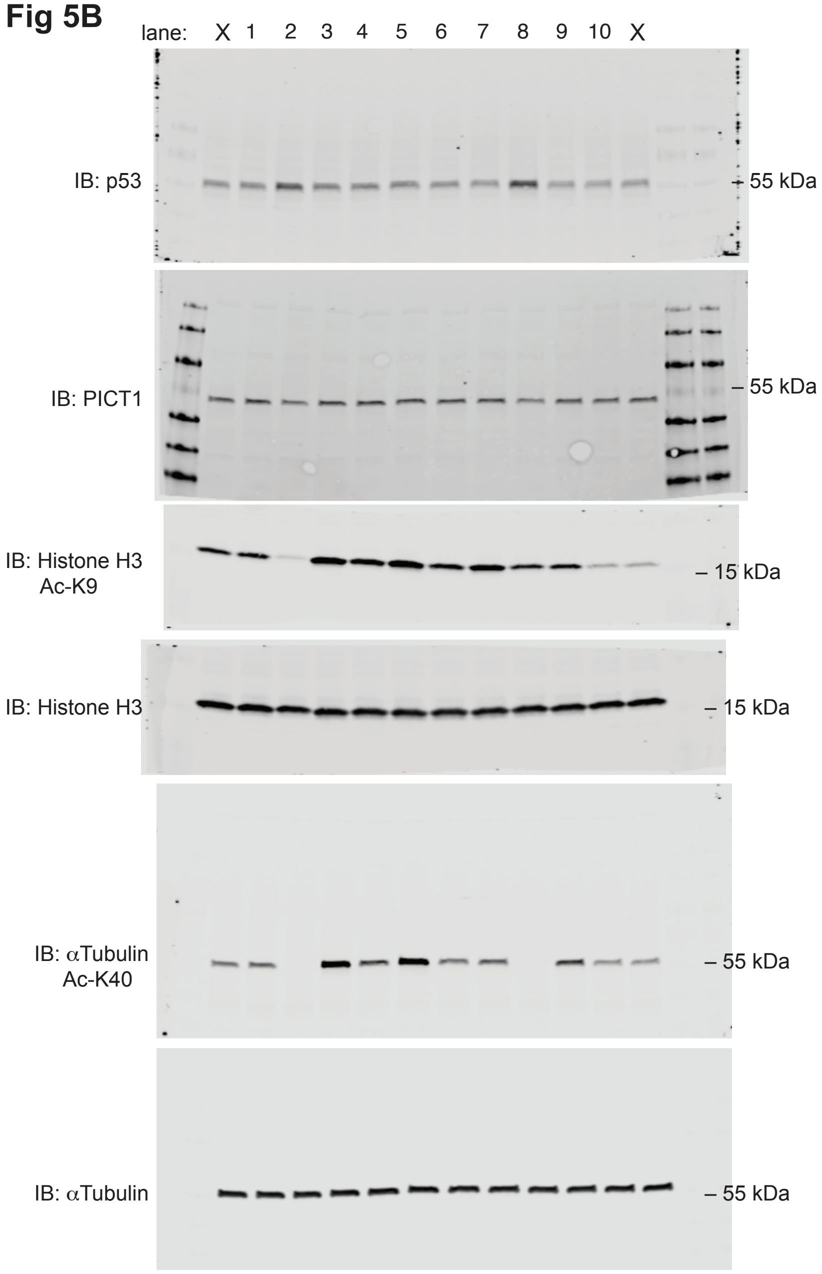

Fig 5C

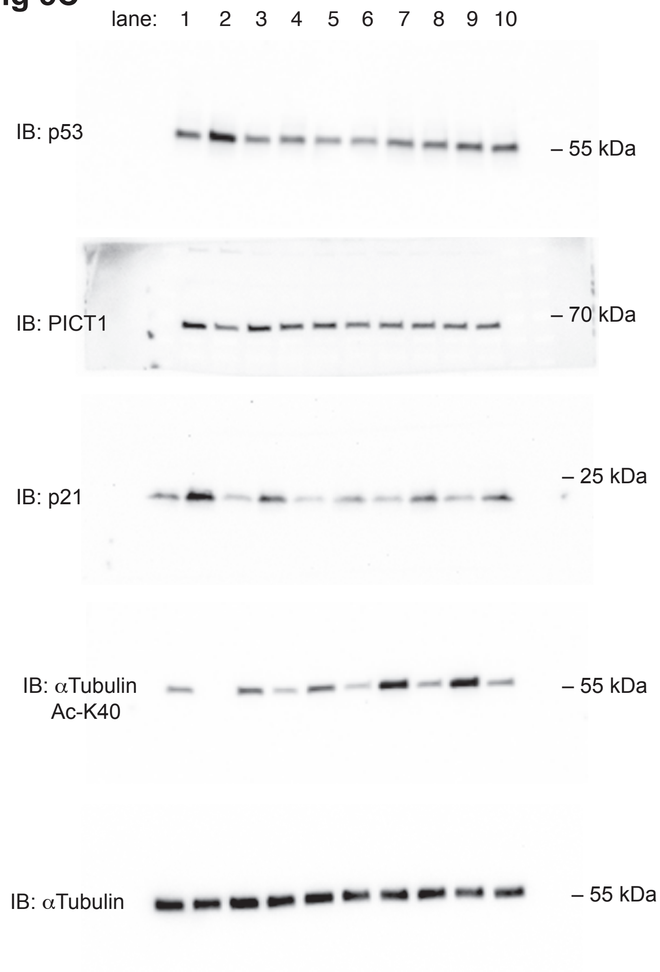

Fig 5F

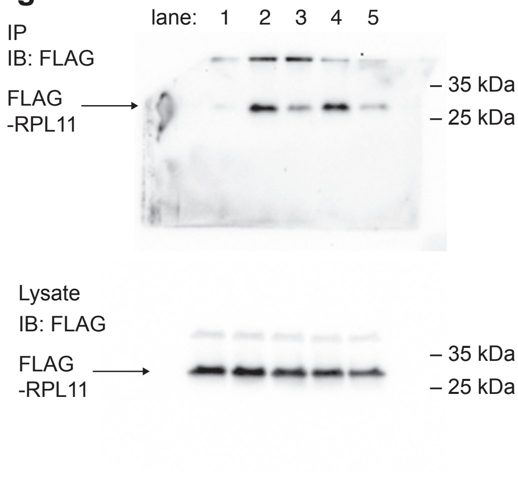

S6A Fig

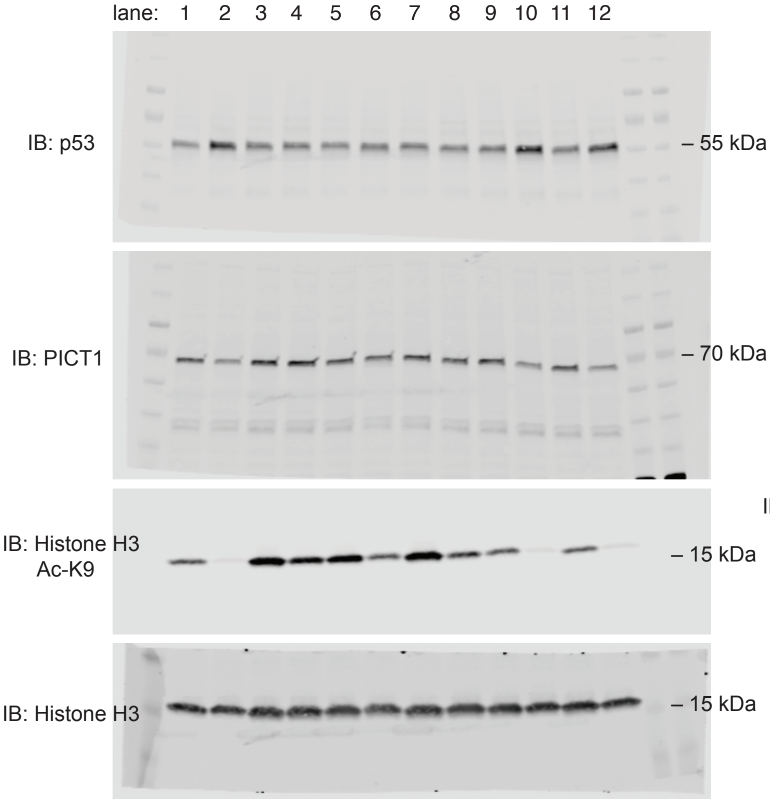

S6B Fig

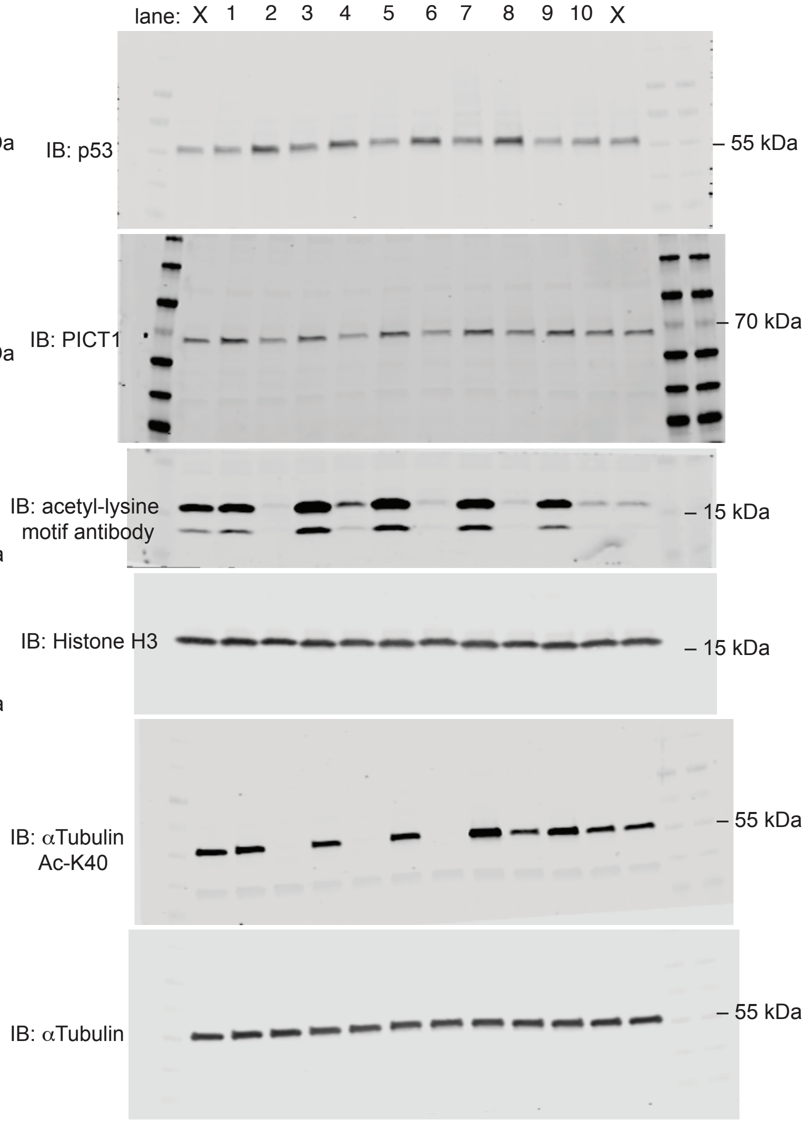

S7C Fig

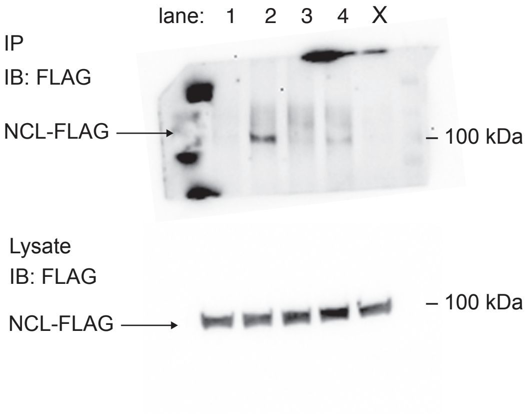

S8A Fig

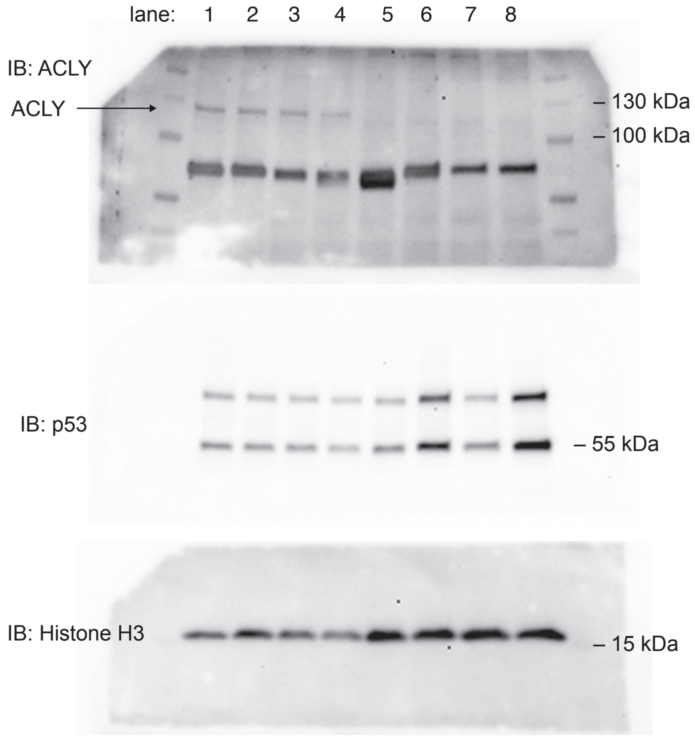

S8B Fig

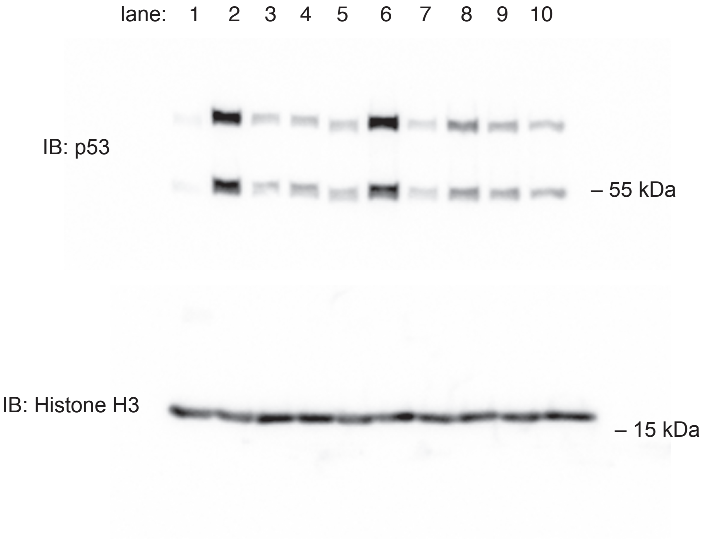

Supplement: S1 Raw images — (PDF) [file pbio.3000981.s013.pdf]
