# Supplementary material for: Acetylation-mediated remodeling of the nucleolus regulates cellular acetyl-CoA responses
Source: PLoS Biol. 2020 Nov 30;18(11):e3000981. doi: 10.1371/journal.pbio.3000981 (PMC7728262; doi:10.1371/journal.pbio.3000981)
Supplement: Attachment — Submitted filename: Response letter .docx [file pbio.3000981.s014.docx]

**Response to reviewers**

First of all, we would like to thank both the reviewers and the editors for reviewing our manuscript and providing us crucial comments to improve our manuscript. Extracted below is the summary of the review by the editor Dr. Ines Alvarez-Garcia from the initial decision letter (received 2020-4-9). As highlighted in red, there are three critical points for this revision; 1) Quantitative validation of acetyl-CoA by another method (i.e. LC-MS), 2) Assessing mTOR signaling, and 3) Re-statement regarding the regulation of liquid-liquid phase separation and molecular dynamics of the nucleolus by acetylation.

Although under the COVID-19 regulation we have experienced limited lab activities and two months delay to begin the experiments for this revision, we have successfully answered all these critical points as described their details below in the responses for each reviewer’s point. Also, we have addressed all reviewers’ comments and, following the reviewers’ suggestions, we have added new data and/or comments in the revised manuscript. Major changes are highlighted in yellow in the revised text (the changes highlighted version).

Editor’s summary

“As you will see, the reviewers are positive of the study and find the conclusions interesting and worth considering for publication. Nevertheless, the reviewers also find that some of the results are not sufficiently supported by the experiments and suggest additional work to strengthen the results. After discussing the reviews with the Academic Editor, we do feel that points by Reviewer 2 about validating the acCoA quantitative data and assessing mTOR signalling are important and should be addressed. Also, as mentioned by both reviewers, the statement 'Overall, these observations demonstrate that class IIa HDACS-mediated deacetylation alters the dynamics of nucleolar proteins,' requires further support or toning down. While addressing the other points would strengthen the conclusions, we do not feel they are essential for publication.”

Reviewers’ comments

Rev. 1:

The manuscript by Dr. Sekine and colleagues, entitled "Acetylation-mediated remodeling of the nucleolus regulates cellular acetyl-CoA responses" clearly demonstrate that decrease in acetyl-CoA levels causes deacetylation of a significant number of nucleolar proteins in a class IIa HDACs deacetylate dependent manner, which compromises rRNA synthesis and activated p53. They have also found that acetyl-CoA decreasing remodels the nucleolar structure via regulation the properties of the LLPS of the nucleolus.

Their data is clean and straightforward, the text is clear, and the conclusions are well supported by the experiments. There are, however, some experimental aspects that should addressed before I would consider this manuscript ready for publication in PLOS BIOLOGY.

Major points:

1)

Fig. 2D and E: I recommend that authors perform the same combination of experiments using ASK-WT cells.

**Response**: We have new data demonstrating alterations in acetylation of histones and tubulin in ASA-WT cells upon acetate withdrawal in revised **S3A Fig** and **Fig 4A**. Interestingly, we observed substantial decreases in acetylation of histone H3 (K9 and K27), H4 (K8) and alpha-tubulin (K40) in ASA-WT cells, although it was to a lesser extent compared with ASA-KO cells. This may be due to observed mild decreases (~20%) in acetyl-CoA levels in ASA-WT cells upon acetate withdrawal (revised **Fig 1E**, mentioned the detail below in “**Response to the reviewer 2 Major point 1**”). It has also been reported that deacetylation of histones can serve as a supply of acetate under certain conditions (**ref 13, 14**), therefore this might be a homeostatic cellular response that stimulates deacetylation in response to a primary decrease in acetate. Since we observed neither remodeling of the nucleolus nor p53 upregulation in ASA-WT cells (revised **Fig 3A-B, S4A-S4B,** **S4F-S4G** **Fig** and **Fig 4A**), this deacetylation appears to be uncoupled to the nucleolar stress response, unlike the coupling observed in ASA-KO cells. We have reflected these comments in the result section (**Page 11, line 6-12, and page 14, line 5-8**).

2)

Fig. 5: Authors have shown that the class IIa HDAC family proteins regulate nucleolar protein deacetylation and nucleolar dynamics by the experiments using specific inhibitors. It would be great if they could specify which of class IIa HDAC family regulates these process by KD experiment using siRNAs.

**Response**: We agree with the reviewer’s concern that identification of the most important class IIa HDAC family member for the nucleolar regulation would be of great interest. However, this task was not easy due to a couple of reasons; 1) the HDAC family members often have functional redundancy (**ref 54**), which may mask an effect of single HDAC knockdown by compensations from other members. 2) siRNA-mediated knockdown experiments need at least 48 or more hours to reduce the target expression. This long duration of HDAC knockdown may affect the basal status of the nucleolus or other cellular conditions. Also, this long time frame may allow the other members to compensate the function of targeted HDAC. In this regard, CRISPR-Cas9-mediated HDAC knockout has the same issue. Indeed, we have conducted siRNA-mediated knockdown of each class IIa HDAC member (HDAC4, 5, 7 and 9) and examined their effects on the reciprocal regulation of p53 and PICT1 upon acetate removal. As shown in “**Figure** **only for the reviewer”**, none of single class IIa HDAC knockdown had significant effect on this regulation nor deacetylation of histones and tubulin. Also, siRNAs for HDAC4, HDAC7 and HDAC9 increased basal p53 levels, which may indicate alterations in the basal cellular conditions. Testing combination of siRNAs for multiple class IIa HDACs would be one way, but, during this limited time of revision, we could not find a combination of siHDACs that completely blocked the reciprocal regulation of p53 and PICT1. Therefore, at this stage, we will refrain from mentioning any particular HDAC in this manuscript and wait for future studies. Nonetheless, we consider that chemical inhibitors are a reasonable way to transiently (only during acetate withdrawal) and broadly (targeting all class IIa HDACs) inhibit class IIa HDACs. We have included these limitations as part of the result section (**Page 16 line 1-2**) and the discussion section (**Page 23 line 14-19**).

3)

Fig. 5G. From this FLAP analysis, authors conclude that class IIa HDACs-mediated deacetylation regulates the dynamics of nucleolar proteins, which are likely associated with change in the LLPS status. The observation is very important findings. However, they have tested only one nucleolar protein, NPM1.

I consider that that they should test at least another nucleolar protein (e.g. FBL, a DFC marker protein, or NCL, a GC marker protein).

**Response**: We understand the both reviewers’ and the academic editor’s concern that the statements regarding the regulation of liquid-liquid phase separation (LLPS) of the nucleolus by acetylation should be toned down as the data in this manuscript is quite limited. The NCL-GFP is so far the only reporter that we could get consistent results for the FRAP analysis, and we would need further time to optimize experimental conditions for other reporters. Therefore, taking all the reviewers’ comments regarding this issue into account, we have re-organized the results and discussion about the FRAP data and the nucleolar LLPS in the revised manuscript. To summarize, we have mentioned the possibility of the regulation of the nucleolar LLPS by acetylation only in the discussion section, clearly mentioning that further analyses will be required to conclude the effect of acetylation on the nucleolar LLPS (**Page 21 line 20- page 22 line 21**). Accordingly, we have minimized the statement for the FRAP experiment in the result section (**Page 17 line 22- page 18 line 12**) and moved the figures to supplemental figure (revised **S7D and S7E Fig**) as suggested by the reviewer 2.

Minor points:

1)

Pages 16-17:

They discuss that "Once acetyl-CoA levels fall, class IIa HDACs deacetylate a significant number of nucleolar proteins. These reactions remodel the properties of the LLPS of the nucleolus, resulting in impairment of rRNA synthesis and induction of the p53-mediated stress responses".

However, we could not rule out the possibility that "Once acetyl-CoA levels fall, class IIa HDACs deacetylate a significant number of nucleolar proteins. These reactions result in impairment of rRNA synthesis, which in turn remodels the properties of the LLPS of the nucleolus and changes the nucleolar structure."

I think they should also consider this possibility.

**Response**: We agree with the reviewer’s suggestion and we have rephrased this sentence as “These reactions result in remodeling of the functional status of the nucleolus including the impairment of rRNA synthesis and the induction of the p53-mediated stress responses.” (**Page 19, line 7-9**).

2)

Page 12, line 23:

"HDM2" instead of "MDM2" because they used human cells (HT1080).

**Response**: Since MDM2 is the official gene symbol for human MDM2 being used in databases such as Entrez Gene, Ensembl and Uniprot, we prefer to use MDM2 in our manuscript. This is also for the consistency of the use of MDM2 in the lists from RNA sequencing and proteomics analysis (**S2 and S3 Tables**). We have included an additional phrase “also known as HDM2” after the first appearance of MDM2 protein (**Page 14, line 15**).

3)

Page 13, line 1:

"protein stability" instead of "protein expression"

**Response**: We have changed the word following the reviewer’s suggestion (**Page 14, line 23-24**). Also, we have newly cited the manuscript (Bursac S et al, PNAS 2012, **ref 48**) that has shown the interdependency of the protein stability between RPL11 and PRL5, which is consistent with our observations.

4)

Page 17, line 1:

"nucleolus" instead of "nucelolus"

**Response**: We have corrected this error.

Rev. 2:

The manuscript by Houston et al. explores the relationship between acetyl-CoA biosynthesis, protein acetylation, and nucleolar function. Specifically, they develop a human fibrosarcoma cell line model in which ATP-citrate lyase (ACLY) is knocked out, and require acetate for proliferation. Acetyl-CoA levels are lower in the knockout cell line upon acetate withdrawal, which the authors use to explore the downstream consequences of depletion of this metabolite. Interestingly, they find that acute depletion of acetyl-CoA does not appear to greatly alter lipid or cholesterol levels; instead, gene expression, proteomic, and acetyl-proteome profiling lead them to identify a depletion in the levels and acetylation of nucleolar proteins. A series of mechanistic follow-up studies are performed, which provides evidence that withdrawal of acetate from ACLY-knockout cells reduces rRNA biogenesis and induces RPL11/RPL5-dependent p53 activation in a manner that can be rescued by HDAC inhibitors, implying a role for protein acetylation in this process. Finally, it is shown acetate also preferntially affects nucleolar liquid-liquid phase separation in ACLY-knockout cells, as assessed by FRAP studies of NCL-mGFP. Overall, the experiments are interesting and well done. If sufficient evidence is provided to implicate acetyl-CoA levels in these phenomena, it will be of substantial interest to the PLOS Biol readership and biological community. However, there are a few experiments missing that must be performed in order to better support some of the major claims of the paper:

1. Quantification of acetyl-CoA: The basis for all of the biological results in the manuscript is that acetate withdrawal from the ACLY knockout cells alters acetyl-CoA levels. This is demonstrated in Figure 1E of the main text, which is quantified on the basis of an HPLC assay examining acetyl-CoA levels from ~2M cells according to the Supplemental. Acetyl-CoA (and CoAs in general) are remarkably difficult metabolites to quantify, suffering both from variable extraction efficiency due to their hydrophobicity as well as lability of their 3'-phosphate group. Based on the figure, the author's measurements of acetyl-CoA and CoA in the acetate-depleted a ratio of ~1:50, which is far beyond what has been measured in previous studies by LC-MS. Since every claim in the paper hinges on the validation of this system to deplete acetyl-CoA, it is absolutely essential that the authors provide an orthogonal measure (i.e. using LC-MS) to demonstrate the dynamics of acetyl-CoA in their system.

**Response**: We have measured acetyl-CoA and CoA levels in ASA-KO cells using LC-MS. First of all, we detected consistent alterations in acetyl-CoA and CoA levels upon acetate withdrawal by the LC-MS method as detected by the HPLC-UV method; approximately 80% decreases in acetyl-CoA levels and 7 to 8-fold increases in CoA levels (revised **Fig 1F**). These results strengthen our observation that acetyl-CoA levels in ASA-KO cells is controlled by supplemented acetate. In the revised **Fig 1E** and **1F**, we have shown the acetyl-CoA and CoA levels as estimated absolute concentrations in a single cell, to directly compare the values obtained from HPLC and LC-MS and also from other studies. Both measurements show the similar range of acetyl-CoA and CoA levels in ASA-KO cells in the basal state (HPLC vs LC-MS; Acetyl-CoA, 2.48 µM vs 4.70 µM; CoA, 16.41 µM vs 5.62 µM). These values were also consistent with the concentrations previously determined by Wellen and Snyder’s group in LN229 cells and IL-3-dependent hepatocyte using LC-MS (**ref 29**). The reciprocal alterations in acetyl-CoA and CoA levels were also observed in the previously reported cellular models of acetyl-CoA depletion (**ref 29, 30**), although the degree was more pronounced in our ASA-KO model. The previous two models [culturing cells in 10 mM or 1 mM glucose containing media (**ref 29**), and genetic deletion of *ACLY* in cultured mouse adipocytes (**ref 30**)] appear to require relatively longer time courses (24 hours or several days) to see a significant decrease in acetyl-CoA levels, which is probably because the acetate-ACSS2 pathway remains intact in these cells. Acetate-withdrawn ASA-KO cells can induce acute acetyl-CoA depletion (within 4 hours after acetate removal) since this condition blocks both the acetyl-CoA producing pathways.

We note that a milder but statistically significant decrease (~20%) in acetyl-CoA concentrations in ASA-WT cells was uncovered, which was not realized in the initial manuscript (**Fig 1E**). This is due to the change of data presentation (from mol/cell number to mol/cell volume) where we found that the cell volume of ASA-WT but not ASA-KO cells was slightly increased upon acetate depletion (the cell volumes were described in the method section). This result indicates that acetyl-CoA levels in ASA-WT cells is partially dependent on supplemented acetate. The observed approximately 20% and 80% contributions of supplemented acetate for acetyl-CoA in whole cell lysate of ASA-WT and ASA-KO cells, respectively, were consistent with previous analysis that determined this contribution using stable isotope labeled acetate in *ACLY* WT and KO MEFs (**ref 27**). Moreover, this partial contribution of acetate to acetyl-CoA appeared to also contribute to acetylation in ASA-WT cells (**S3A Fig**).

We have reflected these comments in the result section (**Page 9 line 9 to page 10 line 4**) and the discussion section (**Page 20 line 5-17**).

2. Given the connection between acetyl-CoA and the nucleolus, it is also essential that the authors also describe what mTOR signaling is doing in these cells. mTor is the most well-known transducer of signals regarding cellular metabolism to ribosome biogenesis. It is certainly conceivable that depletion of acetyl-CoA could cause mTOR inactivation, and previous studies (which should be cited) have linked acetyl-CoA to mTOR's activation state: <https://nam05.safelinks.protection.outlook.com/?url=https%3A%2F%2Fwww.ncbi.nlm.nih.gov%2Fpubmed%2F30197302&amp;data=02%7C01%7Csekiney%40pitt.edu%7C19f02314dfa54bdd856908d7dce33bfc%7C9ef9f489e0a04eeb87cc3a526112fd0d%7C1%7C0%7C637220740837243267&amp;sdata=9E0iF1fh%2Fo70ScVhhGv3BkzegqS3DA%2Bs1%2FW2ppyG75I%3D&amp;reserved=0>. The authors should perform Western blotting examining the status of the mTOR pathway using conventional measures (S6 kinase phosphorylation, etc).

**Response**: We have examined mTORC1 activity upon acetate withdrawal by assessing phosphorylation of S6 kinase. As shown in revised **S5D Fig**, four hours of acetate removal (when the p53 induction was already observed in acetate withdrawn cells) did not suppress the phosphorylation of S6 kinase, while the mTORC1 inhibitor Torin1 was, as expected, effective. In the same experiment, we also monitored the status of LC3, a marker of autophagy induction, since it is also reported that acetyl-CoA depletion induces autophagy (**ref 33, 38, 39**). Similarly, we observed the induction of LC3-II by Torin1 but not by acetate withdrawal. These observations suggest that neither the mTORC1 signaling nor autophagy is affected by acetyl-CoA depletion in ASA-KO cells, at least at this early time point. We have included these comments in the result section (**Page 15, line 8-18**), and cited the above manuscript together with the very recently published manuscript from the same group showing the relation of acetyl-CoA and autophagy (**ref 38, 39**).

3. Page 15: Where on the RPL11 protein does acetylation take place (i.e. what lysine residue)? Based on previous studies, what is the mechanistic hypothesis for how acetylation functionally promotes its activity and impedes activation of p53. Please cite any ltierature precedent.

**Response**: We now identify the TMP195 sensitive acetylation sites of RPL11 as K8 and K67 as listed in the revised **S7B Fig**. As it has been reported that other post transcriptional modifications such as Neddylation (although the exact lysine residues for this modification is unclear) affect the protein stability and nucleolar localization of RPL11 (**ref 71**), acetylation of these lysine residues might also regulate these properties of RPL11. Also, as mentioned in the response below to **Minor point 3**, the low stoichiometry of acetylation may indicate that an entire acetylation state of the nucleolus, rather than the regulation of a single protein or a single lysine residue, may be important for the nucleolar stress response. We have reflected these comments in the result section (**Page 17, line 9-14**) and the discussion (**Page 20, line 19- page 21 line 18**). We have also focused on acetylation of NCL since we detected TMP195-sensitive clustered acetylation (K70, K79, K87, K95, K102, K109, K110, K116, K124 and K132) in its N-terminal region (**S7C Fig**). These lysines are located in the predicted intrinsically disordered region (IDR) of NCL and the IDR is generally an important domain for LLPS. As such, we have included a discussion centering on the possibility that acetylation of the IDR of NCL, a nucleolar hub protein, might affect the phase separation state of the nucleolus by modulating the interaction mode of NCL. These comments have been included in the result (**Page 17, line 14-21**) and the discussion (**Page 22, line 15-21**).

4. I found the liquid-liquid phase separation section of the manuscript to be speculative and uncompelling. The changes in FRAP signal appear to be minor and certainly lack context (i.e. comparison to a stimuli predicted to have a 'major' effect) and the connection to the rest of the manuscript was unclear. This seems like an interesting preliminary experiment for a future paper rather than one essential to the current results, and I would suggest moving it to the SI or removing it to increase focus/clarity,.

**Response**: As also mentioned in the response to the reviewer1’s major point 3), we understand the both reviewers’ and the academic editor’s concern that the statements regarding the regulation of liquid-liquid phase separation (LLPS) of the nucleolus by acetylation should be toned down as the data at this stage is quite limited. Taking all the reviewers’ comments regarding this issue into account, we have re-organized the results and discussion about the FRAP data and the nucleolar LLPS in the revised manuscript. To summarize, we have mentioned the possibility of the regulation of the nucleolar LLPS by acetylation only in the discussion section, clearly mentioning that further analyses will be required to conclude the effect of acetylation on the nucleolar LLPS (**Page 21 line 20- page 22 line 21**). Accordingly, we have minimized the statement for the FRAP experiment in the result section (**Page 17 line 22- page 18 line 12**) and moved the figures to supplemental figure (revised **S7D and S7E Fig**) as suggested.

5. With the above comment (#4), the statement, 'Overall, these observations demonstrate that class IIa HDACS-mediated deacetylation alters the dynamics of nucleolar proteins,' is far too strong a statement based on the data. This should be re-phrased to indicate the current state of the data, 'Overall these observations are consistent with a model in which class IIa HDACS-mediated deacetylation may alter the dynamics of nucleolar proteins, which has the potential to alter the phase state of the nucleolus."

**Response**: With the reorganization of the FRAP section, we have re-phrased the statement as the reviewer suggested (**Page 18, line 10-12**).

Minor points:

1. The strategy described for acetyl-CoA modulation based on ACLY knockout and acetate supplementation is entirely derivative of one that was published by Wellen et al. (Cell Rep, 2016). This was performed for both MEFs as well as glioblastoma cells. The authors mention it, but I found the citation inappropriate as they imply this has not been done previously in human cells by mentioning only the MEF work (page 7). It would not hurt the novelty of this study to more properly acknowledge the field.

**Response**: Following the reviewer’s suggestion, we have included a description for LN299 glioblastoma cells in the introduction section (**Page 5, line 21-24**). The manuscript by Zhao et al (Cell Rep 2016, **ref 27**) has shown that acetate supplementation to ACLY deficient MEFs increased the viability and proliferation, but it has also shown that at least 1 mM acetate did not increase cell proliferation of *ACLY* deficient LN299 cells (Figure S4 of their manuscript). Therefore, we only mentioned *ACLY* deficient MEFs in the context of the result section (**Page 8, line 17-18**).

2. Consistent with the discussion of acetyl-CoA/CoA quantification above, the authors should provide HPLC chromatograms and calibration curves for the metabolite quantifications given in Figure 1E. This is important as again, the entire manuscript hinges on the quality of these quantificaitons.

**Response**: In the revised **S2 Fig**, we have newly included HPLC UV chromatograms of cell lysates from a representative experiment (among three independent experiments) for the acetyl-CoA and CoA quantification (**Fig 1E**) and HPLC UV chromatograms of standard acetyl-CoA and CoA (not for standard curves) run in the same experiment.

3. The detection of dynamically acetylated ribosomal proteins is interesting; however, proteomic datasets are biased towards the detection of ribosomal proteins as they are the most abundant proteins in cells, and they may be modified at low stoichometries that are unlikely to affect their activity

(<https://nam05.safelinks.protection.outlook.com/?url=https%3A%2F%2Fwww.nature.com%2Farticles%2Fs41467-019-09024-0&amp;data=02%7C01%7Csekiney%40pitt.edu%7C19f02314dfa54bdd856908d7dce33bfc%7C9ef9f489e0a04eeb87cc3a526112fd0d%7C1%7C0%7C637220740837243267&amp;sdata=YAWQmwtqgBYYOJvDqNm%2Bgt2gGEwv5TU7wkZx7xWy2DQ%3D&amp;reserved=0>). The authors should comment on this issue, and also explicitly describe if any of their acetyl-CoA/dynamic acetylations are found at 'high stoichiometry sites as described by Hansen et al. (referenced in link above)

**Response**: We appreciate that the reviewer has drawn our attention to this important manuscript. We have checked stoichiometries of TMP195 sensitive acetylation listed in **S7B Fig**. According to their data, the stoichiometries of acetylation (including RPL11 and other ribosomal proteins) was very low (mostly around 0.05–0.5%). As the authors of this manuscript have also mentioned in their manuscript, the low stoichiometry of acetylation does not necessarily mean a lack of the effect of acetylation. This rather implicates that spatial and temporal regulation of acetylation may be important for modulating a biological process by acetylation. Also, it is likely that rather than acetylation of a single residue or a single protein, an entire balance of multiple acetylation residues may be important. We have reflected these comments in the discussion (**Page 21, line 7-18**).

Regarding the bias due to the abundance of ribosomal protein, we have detected not only ribosomal proteins but many other proteins as TMP195 sensitive proteins such as molecules involved in cell-cell adhesion, mRNA splicing, rRNA processing, translational initiation, nucleosome assembly, and regulation of transcription. We have included this GO analysis in revised **S7A Fig** and in the result section (**Page 17, line 6-7**).

Lastly, we have summarized the manuscripts that we have newly cited in the revised manuscript. The research field of acetyl-CoA regulation, as well as that of the nucleolar stress response, has been growing rapidly and several important papers have been published very recently that make the introduction section of our manuscript more fruitful and strengthen our result and discussion. Therefore, we have cited these papers in the revised manuscript as much as possible, together with several papers that we had overlooked in the initial manuscript and papers that were suggested by the reviewers. These include **refs 11, 12, 13, 14, 15, 16, 24, 25** for acetate sources and acetate contributions to gene regulation, **refs 38, 39** for acetyl-CoA and mTOR signaling and autophagy, **refs 48, 49, 64, 71, 87** for the nucleolar stress response, **ref 52** for mTORC1 signaling for ribosome biogenesis, **ref 68** for the stoichiometry of acetylation, **ref 78** for effect of acetylation on the LLPS of chromatin. Accordingly, we have partly revised the introduction section (**Page 5, line 8-12, line 17-24, Page 6, line 1-12, line 16-17**).

We also note that Steven J. Mullet has been included in the author list for his contribution to the acetyl-CoA and CoA measurement using LC-MS.
