# Supplementary material for: Acetylation-mediated remodeling of the nucleolus regulates cellular acetyl-CoA responses
Source: PLoS Biol. 2020 Nov 30;18(11):e3000981. doi: 10.1371/journal.pbio.3000981 (PMC7728262; doi:10.1371/journal.pbio.3000981)
Supplement: Attachment — Submitted filename: Response letter 2.docx [file pbio.3000981.s015.docx]

**Response to reviewers**

Reviewer's comments

Rev. 1:

The authors improved manuscript in response to my comment. Though they have not done all the experiments that I requested, their response to my comments is reasonable. Therefore, I consider that this manuscript should be accepted.

Only one minor point:

LMK235 instead of LMK234 (page 18, line 8).

**Response**: We have corrected this error.
